# A Pilot Randomized Placebo Controlled Trial of Electroacupuncture for Women with Pure Stress Urinary Incontinence

**DOI:** 10.1371/journal.pone.0150821

**Published:** 2016-03-09

**Authors:** Huanfang Xu, Baoyan Liu, Jiani Wu, Ruosang Du, Xiaoxu Liu, Jinna Yu, Zhishun Liu

**Affiliations:** 1 Department of acupuncture and moxibustion, Guang’anmen Hospital, China Academy of Chinese Medical Sciences, Beijing, China; 2 School of Acupuncture-Moxibustion and Tuina, Beijing University of Chinese Medicine, Beijing, China; Stavanger University Hospital, NORWAY

## Abstract

**Background:**

Acupuncture is a potential conservative therapy for women with stress urinary incontinence (SUI). There is limited evidence to support its effectiveness due to the poor quality of existing studies.

**Methods:**

We performed a pilot randomized, controlled trial to preliminarily assess the efficacy of electroacupuncture (EA) in women with pure SUI. A total of 80 women with pure SUI were randomly assigned to receive EA with deep needling at BL33 and BL35 (n = 40) or sham EA with non-penetrating needling at sham acupoints (n = 40) three sessions per week for 6 weeks. The women were followed for 24 weeks. The primary outcome was the change from baseline in the amount of urine leakage measured by a 1-hour pad test after 6 weeks. The secondary outcomes included the 72-hour incontinence episode frequency (IEF), International Consultation on Incontinence Questionnaire-Short Form (ICIQ-SF) score, and patient self-evaluation of therapeutic effect. Adverse events (AEs) were monitored throughout the trial.

**Results:**

The median decrease from baseline of urine leakage measured by the 1-hour pad test was 2.5 g [interquartile range (IQR): 1.80–14.6 in the EA group, which was greater than the median decrease of 0.05 g (IQR: -2.80–+0.50) in the sham EA group after 6 weeks (p<0.01). The differences between groups in the decrease from baseline of 72-hour IEF became statistically significant at week 30 with a median decrease of 3.25 g (IQR: 1.25–5.69) in the EA group, and a median decrease of 1.00 g (IQR: -0.69–+2.88) in the sham EA group (p = 0.01). The participants in the EA group showed greater decreases in ICIQ-SF score and higher ratings in the help they received from the treatment than those in the sham EA group at weeks 6,18 and 30 (all p<0.05). No obvious AEs were observed in either group.

**Conclusion:**

EA may effectively and safely relieve urinary incontinence symptoms and improve quality of life in women with pure SUI. EA demonstrated more than a placebo effect. Since this is a pilot study, results should be interpreted with caution.

**Trial Registration:**

ClinicalTrials.gov NCT02445573.

## Introduction

Stress urinary incontinence (SUI), defined as an involuntary loss of urine on physical exertion, sneezing, or coughing [1], is a common health problem among women worldwide. It accounts for approximately 50% of urinary incontinence (UI) with a prevalence of 24.8% (95% CI: 23.4–26.3) in the United States [2] and 18.9% in China [3]. SUI negatively affects patients’ physical health and social and psychological well-being [4, 5], with harm greater than major chronic conditions including diabetes, hyperlipidemia, and chronic kidney disease [6]. Among conservative therapies, pelvic floor muscle training (PFMT) is generally regarded as the first line management for SUI. However, a length of at least three months is recommended for the practice of PFMT to see a major change [7–9], and the adherence rate was observed to be correlated negatively with time [10]. Therefore, an effective treatment that is quick and easy to comply with is still needed for the management of SUI. Acupuncture, which has been practiced for over 2,500 years in China, has gained increasing international attention in healthcare. Acupuncture has been reported to be useful for treating SUI, with a total effective rate up to 90% within approximately 1 month [11, 12]. However, our systematic review showed that acupuncture was effective for SUI with limited evidence [13] due to the poor quality of the included randomized controlled trials (RCTs). We therefore conducted a pilot randomized placebo controlled trial to preliminarily assess the efficacy of EA in women with pure SUI.

## Methods

### Study design

This investigation was a randomized, placebo-controlled study. It was conducted between December 2012 and June 2014. The study was conducted in accordance with the Declaration of Helsinki, and the protocol was reviewed and approved by the Ethics Committee of Guang’anmen hospital of China Academy of Chinese Medical Sciences (CACMS). This study was retrospectively registered on ClinicalTrials.gov (Identifier: NCT02445573). The authors confirm that all ongoing and related trials for this intervention are registered.

### Participants

Participants were recruited through posters and specialist recommendations in Guang’anmen hospital of CACMS, Beijing, China. All participants provided written informed consent after the trial procedures were fully explained. Potential participants were instructed to finish the laboratory examinations (urine routine, urine flow rate and the residual urine), complete a 72-hour bladder diary and ICIQ-SF, and take a 1 hour pad test by research assistants during 1-week baseline assessment to determine eligibility. Eligible women were aged 40 to 75 years, and met the following clinical diagnosis recommendations of SUI by the International Consultation on Urological Diseases [7]: involuntary urine leakage on effort, exertion, sneezing or coughing, which stopped when the stress ends; visible involuntary leakage from the urethra synchronous with increased abdominal pressure, or a pad weight gain >1 g in 1-hour pad test; and without symptoms of urinary frequency and urgency. Women were excluded from the study if they met any of the following criteria: other type of UI (urge, mixed, or overflow UI, etc); symptomatic urinary tract infection; ever received UI or pelvic surgery; severe pelvic organ prolapse ≥ degree 2; residual urinary volume >30 ml; maximum flow rate ≤ 20 ml/s; limited in walking, stairs climbing and running; receiving specialized treatment for SUI (mainly SUI medications such as duloxetine, PFMT, feedback therapy, electrical or magnetic stimulation via pelvic floor, vagina or anus, and transcutaneous electrical nerve stimulation to pelvic floor), or use of medicine affecting bladder function; serious cardiovascular, cerebral, liver, kidney, or psychiatric disease, diabetes, multiple system atrophy, injury of cauda equina, or myeleterosis; being pregnant or breastfeeding; with cardiac pacemaker, metal allergy or severe needle phobia; or unlikely to give written informed consent.

### Randomization and Blinding

Eligible participants were randomly assigned to the EA group or the sham EA group in a 1:1 ratio via a central randomization system operated by the clinical evaluation center of CACMS. The randomization scheme was generated using PROC PLAN of Statistical Analysis System (SAS, version 9.4, SAS Institute, Cary, NC, USA). The random number and group division and were obtained by acupuncturists through the phone or the web. The concealment of treatment allocation was implemented with central randomization. In this trial, the participants, outcome assessors and statisticians were blinded to treatment allocation. Subject blinding was achieved via the aid of adhesive pads used in both groups, placebo needle with a blunt tip and sham EA electrode lines. The placebo needle used in this trial has already been tested to show a good subject blinding effect [14]. The sham electrode line was identical in appearance with the real electrode, however, its inner metal wire was cut off. Therefore, even if the EA apparatus showed a power-on state, there was no current.

### Intervention

There were 2 full-time certified acupuncturists with a clinical acupuncture experience of ≥2 years who were responsible for the manipulation of EA and sham EA. Disposable acupuncture needles (size 0.30×75 mm), pragmatic placebo needle (size 0.30×25 mm) and the SDZ-V EA apparatus (all were Hwato Brand, Suzhou Medical Appliance Factory, Suzhou, China) were used in this trial.

In the EA group, the acupoints of bilateral BL33 (Zhongliao) and BL35 (Huiyang), located according to the World Health Organization Standardized Acupuncture Points Location, [15] were used. BL33 is on the third posterior sacral foremen. BL35 is 0.5 cun (an essential component of traditional point location methods used in acupuncture [16]) lateral to the tip of the coccyx. When acupuncturing, adhesive pads were first pasted on acupoints after sterilization in either group. In the EA group, the participants were needled at bilateral BL33 at an angle of 30 to 45 degree inward and downward, and at bilateral BL35 slightly toward upside and outside, to a depth of 50 to 60 mm using acupuncture needles of size 0.30×75 mm. In the sham EA group, participants were needled at sham BL33 and sham BL35, which were approximately 20mm lateral to BL33 and BL35, respectively, with blunt needle tips piercing adhesive pads and not piercing the surface of the skin, using placebo needles of size 0.30×25 mm. Needles were then lifted, thrusted and twirled evenly 3 times to achieve deqi (also termed as needle sensation, which is a composite of unique sensations interpreted as the flow of qi induced by acupuncture, and is believed to be essential for good clinical effect [17]). To implement a standardized operation among various participants, needle manipulation would be no more performed even if the required manipulation failed to achieve deqi. Paired electrodes of the EA apparatus were attached transversely to bilateral BL33 and BL35 (using real electrodes), respectively, or to bilateral sham BL33 and sham BL35 (using sham electrodes) respectively, with a continuous wave of 50 Hz and a current intensity of 1–5 mA for 30 min. The participants were treated with EA or sham EA 3 sessions per week on alternate days for 6 successive weeks.

To improve the effect of blindness, participants were informed that the trial intended to compare the effects of two EA methods for pure SUI. One was traditional EA using conventional acupoints and electric current, and the other was non-traditional EA by needling non-traditional acupoints with a weak electric current. The inform consent form was approved by our ethics committee. The participants were discouraged from any other specialized treatments of SUI, mainly SUI medications such as duloxetine, PFMT, feedback therapy, electrical or magnetic stimulation via pelvic floor, vagina or anus, and transcutaneous electrical nerve stimulation to the pelvic floor. During the treatment period, if a participant was menstruating, the treatment was postponed until menstruation ended. The length of delay was not included in the treatment period. It took 32 weeks in total for a patient to complete the trial: 1 week of screening, 1 week of baseline assessment, 6 weeks of treatment, and 24 weeks of follow up.

### Outcome Measures

The primary outcome was the change from baseline of urine leakage after 6 weeks measured by the 1-hour pad test. The 1-hour pad test was performed according to the International Continence Society instructions [18]. Patients were instructed to void 2 hours before the pad test. On arrival, they received a pre-weighed pad and were asked to sit and drink 500 ml sodium-free water in 15 minutes. Next, they were instructed to walk for 30 minutes, including going up and down 24 steps. On returning to the clinic, they were instructed to perform several activities, including standing and sitting 10 times, coughing vigorously 10 times, running on the spot for 1 minute, picking up a coin from the floor 5 times, and putting their hands under water for 1 minute. After the activities were completed, the pad was reweighed to measure the amount of urinary leakage.

The secondary outcomes included change from baseline of the mean 72-hour incontinence episode frequency (IEF) during weeks 1–6, weeks 15–18 and weeks 27–30, and change from baseline of the total ICIQ-SF scores and patient self-evaluation of therapeutic effect at weeks 6, 18 and 30.

The data of the IEF were from the 72-hour bladder diary recorded by participants at baseline (week 0), and during the treatment period (weeks 2, 4 and 6) and follow-up period (weeks 15–18 and weeks 27–30). In the bladder diary, the participant recorded in detail the time and frequency of UI, activity that occurred at the time of leak, and the type and volume of liquid intake. In case that a participant was in her menstrual cycle or suffering from a severe cough during an assessment week, she was instructed to not record in the bladder diary until the end of the cycle or the recovery of cough. The ICIQ-SF was a brief and robust measure for evaluating the symptoms and impact of urinary incontinence [19]. A Chinese version of ICIQ-SF had been developed and successfully validated [20]. It was used to assess the influence of UI on quality of life during the previous 4 weeks. It contained three items on frequency, amount of leakage, and overall impact on quality of life and a fourth, non-scored item that assessed the type of incontinence. Scoring was additive (0–21), with higher values indicating increased severity. The total score of week 6 was the average of the sum of weeks 4 and 6. Patient self-evaluation of the therapeutic effect was measured by a 4-point scale to rate the extent of help that the participants thought they received from treatment (0, no help; 1, little help; 2, moderate help; 3, great help).

### Safety Monitoring

Acupuncture is relatively safe if practiced by competent practitioners [21]. Therefore, our trial only recorded and evaluated the serious adverse events (SAEs) and the AEs related to EA, including local pain from needling, slight bleeding or hematoma, persistent pain after EA, discomfort after EA (remaining uncomfortable needle sensation, fatigue, etc), stuck needle, fainting during acupuncture, broken needle, and infection. Participants were instructed to report AEs throughout the study by contacting the outcome assessor at each visit or by telephone.

Pain or discomfort caused by acupuncture (if any) was assessed using a 10-point visual analogue scale (VAS, 0 indicates no pain/discomfort, and 10 indicates the severest pain/discomfort). Given that acupuncture was a minimally invasive therapy inevitably causing pain, acupuncture pain with a spontaneous remission within 30 min after acupuncture was not regarded as an AE. Other common AEs that were obviously not related to acupuncture (such as developing a cold) were neglected. The rate of AEs were calculated using the data of patients with ≥1 AEs.

### Sample Size and Statistical Analysis

Assuming a two-sided alpha of 0.05, power of 90%, and a 20% drop-out rate, a sample size of 36 would be needed for each group to detect a between-group mean difference of 1.46 g (SD 1.74) in the reduction from baseline of urine leakage as measured by the 1-hour pad test according to the results of our previous SUI study comparing deep EA with shallow EA [22]. We expanded the sample size to 80 cases (40 cases per group) to increase the reliability of the study.

We performed a statistical analysis based on the intention-to-treat principle. All patients accepting randomization were included in the analysis. Missing data were completed by the last observed value. Continuous data were presented with mean and standard deviation [M (SD)] if they were normally distributed or with median and interquartile ranges [Median (IQR)] if they were abnormally distributed. Categorical variables were expressed as numbers and percentages. Student’s t tests or Mann-Whitney U tests were used to compare continuous variables; chi-square tests, Fisher’s exact tests or Kruskal-Wallis H tests were used to compare categorical variables, as appropriate. For measures collected at two time points, paired t-tests or Wilcoxon signed rank tests were used as appropriate. A statistically significant difference was set at P<0.05. All statistical analyses were performed using SPSS statistical software (version 20.0, International Business Machines Corporation, China).

## Results

### Study population

**Fig 1:**
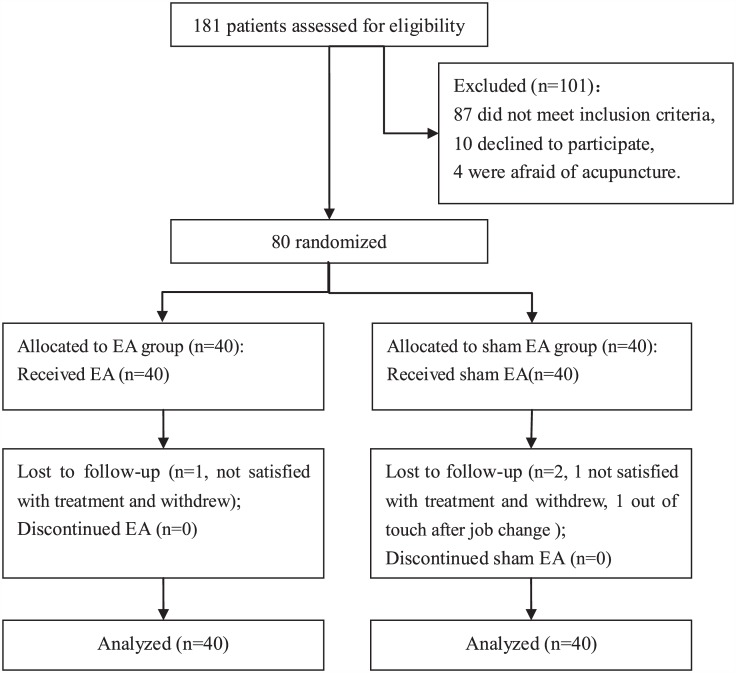
Flow diagram of participants. The flow chart of this study according to the CONSORT Statement.

A total of 181 women with UI were invited to participate in the study, of whom 101 were excluded, and 80 were eligible and randomly assigned to the EA group (n = 40) or the sham EA group (n = 40). All participants completed 6 weeks of EA or sham EA, but 3 participants were lost to follow up. Details are listed in Fig 1.

There were no statistically significant differences in baseline demographic and clinical characteristics between the two groups except that more participants in the sham EA groups suffered from severe SUI rated by the amount of urine leakage measured by the 1-hour pad test [23] (Table 1).

**Table 1:** Baseline Demographic and Clinical Characteristics of the Study Population.

Characteristic	EA Group (n = 40)	Sham EA Group (n = 40)
Age, year^†^	59.05 (7.91)	57.97 (8.42)
Educational level- No. (%)		
Primary education or below	2 (5.00)	5 (12.50)
Secondary education	30 (75.00)	21 (52.50)
Tertiary education	8 (20.00)	14 (35.00)
Childbearing- No. (%)		
Yes	40 (100)	38 (95.00)
No	0	2 (5.00)
Number of births- No. (%)		
0	0	2 (5.00)
1	25 (62.50)	25 (62.50)
2	9 (22.50)	7 (17.50)
≥3	6 (15.00)	6 (15.00)
Initial childbearing age^†^	24.65 (2.94)	23.71 (2.84)
Number of vaginal deliveries^‡^	1.00 (1.00–2.00)	1.00 (1.00–2.00)
Number of cesarean	0	0
Menopause- No. (%)		
Yes	31 (77.5)	30 (75.00)
No	9 (22.5)	10 (25.00)
Hysterectomy- No. (%)		
Yes	3 (7.5)	2 (5.00)
No	37 (92.5)	38 (95.00)
BMI^‡^	23.44 (22.09–25.53)	22.70 (21.47–24.28)
Duration of disease, year ^‡^	5.04 (2.54–8.81)	5.00 (2.37–8.07)
Ever received SUI treatment- No. (%)		
Yes	6 (15.0)	2 (5.0)
No	34 (85.0)	38 (95.0)
Severity of SUI rated by the amount of urine leakage measured by the 1-hour pad test*- No. (%)		
Mild (1.1–9.9 g)	27 (67.5)	27 (67.5)
Moderate (10–49.9 g)	12 (30.0)	6 (15.0)
Severe (≥50 g)	1(2.5)	7 (17.5)
Urine leakage measured by the 1-hour pad test^‡^	5.30 (2.33–16.00)	4.60 (2.30–17.70)
72-hour IEF^‡^	4.00 (2.00–6.75)	5.00 (1.25–9.00)
ICIQ-SF score^†^	9.08 (4.24)	8.80 (4.54)

BMI, body mass index, is the weight in kilograms divided by the square of the height in meters.

^†^, Mean (SD);

^‡^, Median (IOR);

*, p<0.05.

### Effectiveness

The decrease from baseline of urine leakage measured by the 1-hour pad test after 6 weeks of treatment was significant in the EA group (p<0.001) with a median of 2.50 g (IQR: 1.80–14.60), which was equal to a median decrease of 84% (IQR: 60%–93%). There was negligible change in the sham EA group (p = 0.54), with a median of 0.20 g (IQR: -2.08 to 3.83), an equivalent of a 5% decrease (IQR: -28% to 50%). The decrease from baseline of urine leakage after 6 weeks was greater in the EA group than that in the sham EA group (p<0.01). Details are listed in Table 2.

**Table 2:** Primary and secondary outcomes of the interventions.

Outcome	EA Group (n = 40)	Sham-EA Group (n = 40)	*P* Value^§^
**Urine leakage measured by the 1-hour pad test**^‡^^ǂ^			
Week 6	0.90 (0.40–1.68)**	3.85 (2.13–17.25)	p<0.001
Week 6- change from baseline	2.50 (1.80–14.60)	0.05 (-0.28–+0.50)	p = 0.001
**72-hour IEF**^‡^^ǂ^			
Week 6- change from baseline	2.00 (0.42–3.25)	0.67 (-0.59–+2.25)	p = 0.09
Week 18- change from baseline	3.00 (1.06–5.00)	2.00 (0–5.19)	p = 0.19
Week 30- change from baseline	3.25 (1.25–5.69)	1.00 (-0.69–2.88)	p = 0.01
**ICIQ-SF score**^ǂ^			
Week 6- change from baseline^‡^	1.75 (0–5.63)	0 (-1–+1)	p = 0.001
Week 18- change from baseline^†^	5.60 (4.19)	2.13 (4.20)	p<0.001
Week 30- change from baseline^†^	5.89 (4.29)	1.48 (3.39)	p<0.001
**Patient self- evaluation of therapeutic effect**-No. (%)			
Week 6	No: 0; Little: 7 (17.5%); Moderate: 11 (27.5%); Great: 22 (55.0%)	No: 15 (37.5%); Little: 14 (35.0%); Moderate: 4 (10.0%); Great: 7 (17.5%)	p<0.001
Week 18	No: 1 (2.5%); Little: 5 (12.5%); Moderate: 9 (22.5%); Great: 25 (31.3%)	No: 15 (37.5%); Little: 8 (20.0%); Moderate: 5 (12.5%); Great: 12 (15.0%)	p<0.001
Week 30	No: 1 (2.5%); Little: 4 (10.0%); Moderate: 8 (20.0%); Great: 27 (67.5%)	No: 17 (42.5%); Little: 11 (27.5%); Moderate: 3 (7.5%); Great: 9 (22.5%)	p<0.001

^ǂ^, for the results of change from baseline, positive values indicate improvement from baseline, whereas negative values indicate aggravation;

^§^, indicates p value for the between-group comparison;

**, p<0.01 for the comparison with baseline;

^‡^, Median (IOR);

^†^, Mean (SD).

Compared with baseline, the mean 72-hour IEF decreased significantly at weeks 6, 18 and 30 in the EA group (all p<0.05) and at weeks 18 and 30 in the sham EA group (both p<0.01). However, a significant difference in the change from baseline of 72-hour IEF between the groups was only observed at week 30 (p = 0.01): a median decrease of 3.25 sessions (IQR: 1.25–5.69) in the EA group, which was greater than the median of 1.00 session (IQR: -0.69 to 2.88) in the sham EA group (Table 2).

The participants reported substantial improvements in total ICIQ-SF score at weeks 6, 18 and 30 in the EA group (all p<0.05) and at week 18 in the sham EA group (p = 0.02) compared to baseline. There were significant between group differences in total ICIQ-SF score and patient self-evaluation of therapeutic effect at weeks 6, 18 and 30, (all p<0.05) (i.e., participants in the EA group showed a greater decrease in the ICIQ-SF score and a higher rating in the help they received from the treatment than those in the sham EA group) (Table 2).

### Adverse events

No SAEs and few EA related AEs were observed in our trial. There was no significant difference between the groups in the incidence of AEs, with 7.50% (3/40) in the EA group (2 cases of hematoma at the needling site, and 1 case of persistent pain after EA of vas = 5), and 5.00% (2/40) in the sham EA group (persistent pain and fatigue, both vas = 4).

## Discussion

The results of this randomized, placebo controlled trial showed that EA alleviated symptoms of urinary incontinence and improved quality of life in women with pure SUI. Its effect was superior to sham EA, which indicated that the effect of EA was not a placebo effect but a specific treatment effect.

Pad tests enable an objective measurement of urine loss, among which the 1-hour pad test was recommended by the International Incontinence Society (ICS)[18]. To our knowledge, there is no literature on the effect of acupuncture for SUI measured by the 1-hour pad test. In the literatures on PFMT, a mean decrease of 5.11 g (SD 7.29) was found in an 8-week PFMT trial [24], and a mean decrease of 7.9 g (SD 12.1) was found in a 12-week PFMT trial [25]. It seemed that either a decrease in urine leakage treated by PFMT was larger than a median decline of 2.50 g (IQR: 1.80–14.60) of urine leakage treated by EA in our trial. However, this finding did not indicate the inferiority of EA given the incomparable baseline levels of urine leakage between the PFMT literature and our findings. It was reported that a decrease of ≥50% in urine loss qualified as clinical improvement [26–28]. In this trial, the participants in the sham EA group experienced no significant changes in urine leakage as measured by the 1-hour pad test after 6 weeks compared with baseline. In contrast, the participants in the EA group reported a significant drop in urine leakage after 6 weeks compared with baseline and a greater decrease from baseline than those in the sham EA group. Moreover, the improvement in the EA group reached the criterion of clinical effectiveness with a median decline of 84% (IQR 60%–93%), which far exceeded 50%. Briefly, the effect of EA was more than a placebo effect, and it can be used to improve urine leakage as measured by the 1-hour pad test in clinical practice.

Although improvements were observed for both EA and sham EA, the differences between the groups in the decrease from baseline of 72-hour IEF were not statistically significant until week 30. This finding implied a sustained effect of EA after stopping treatment. The median decrease from baseline of 72-hour IEF in the EA group at week 30 reached a median of 3.25 sessions (IQR 1.25–5.69), similar to decreases from baseline of the weekly mean IEF of 7.6 sessions (95% CI 5.7–5.9) after 3-month internet based PFMT [29] and 7.25 sessions after 8-week treatment of duloxetine [30]from a perspective of daily decrease. It was reported that patients appeared to recognize important clinical value of IEF at reductions of approximately 50%[31]. In our trial, the proportions of patients with a reduction in 72-hour IEF of >50% were approximately 70% at week 18 and 77.5% at week 30, similar to the 69.8% with PFMT [29].

The advantages of EA over sham EA in the improvement of quality of life based on ICIQ-SF scores were noted from week 6 through week 18 to week 30, indicating good short-term and long-term specific effects of EA. In Tomoe Hirakawa’s study, a reduction of approximately 3.7 in the scores were observed after 12 weeks of PFMT treatment [32]. In our study, the reduction in ICIQ-SF score was lower than that of PFMT [32] after 6 weeks of EA treatment with a median of 1.75 scores but surpassed that of PFMT [32]by a median reduction of 5.6 and 5.89 at weeks 18 and 30, respectively. In regard to clinical significance, a decrease in ICIQ-SF score to 5 or less was defined as a cure [33]. In our trial, the participants who received EA began to gain clinical recovery from week 18 to week 30 with median ICIQ-SF scores less than 5, whereas participants in the sham EA group did not reach the standard of clinical recovery at any assessment period. This result reconfirmed the effect superiority of EA over sham EA.

The effect superiority of EA over sham EA was also recognized by participants. Approximately 53.8% to 87.5% of participants in the EA group thought they received no less than moderate help from EA, in contrast to the range of 27.5% to 30% in the sham EA group. Patient satisfaction of EA in our trial was similar to that of PFMT: 84.7% (161/190) of patients with SUI treated by home intravaginal electrical stimulation of the pelvic floor [34] and 84.8% of patients with SUI treated by internet-based PFMT [29].

Acupuncture is relatively safe if practiced by competent practitioners; no SAEs were reported (95% CI: 0 to 1.1 per 10,000 treatments), and there was a low rate of 1.3 per 1,000 treatments (95% CI: 0.9 to 1.7) for AEs [21]. The results of our trial provided new evidence on the safety of EA application.

In this study, BL33 and BL35 were deeply needled to treat SUI. The selection of these two acupoints was based on both meridian theory in traditional Chinese medicine (TCM) and the innervations of the urinary system in western medicine. In TCM, SUI is related to the dysfunction of the bladder. According to the meridian theory, an acupoint has its own therapeutic feature due to its particular location and pertaining meridian. Therefore, acupoints pertaining to the bladder meridian and located on the lumbosacral region (adjacent to the bladder) are preferred in acupuncture for SUI. BL33 and BL35 were among those eligible acupoints. The pathophysiology of SUI is closely related to insufficient functioning of the urethral sphincter and urethral hypermobility due to weakened pelvic floor muscles [35]. The parasympathetic control of the bladder musculature, the contraction of which causes bladder emptying, originates with neurons in the sacral spinal cord segments S2 to S4. The striated muscle of the external urethral sphincter is innervated by motor neurons that originate in Onuf’s nucleus (located within the sacral spinal cord) and travel via the pudendal nerve. BL33 and BL35 are on the course of the posterior branch of the S_3_ sacral nerve and the pudendal nerve, respectively, so deep electroacupuncture on BL33 and BL35 can stimulate the the third sacral nerve and the pudendal nerve. Direct modulation of the S_2_-S_4_ sacral nerve activity influences the behavior of the pelvic floor, lower urinary track and urinary sphincters by sacral neuromodulation [36, 37] and posterior tibial nerve stimulation [38]. Therefore, we insist that the neuromodulation for bladder control is the possible mechanism for the effect of EA on SUI. However, it is important to note that, due to the use of non-penetrating needling on sham acupoints, the specific point effects of BL33 and BL35 need to be validated in further research.

There were limitations in our study. An ideal placebo control was designed to yield no or minimum curative effect. In this trial, we used sham EA as placebo control. The sham EA was consisted by a placebo needle and a deactivated EA apparatus. The placebo needle was without skin penetration, and its patient blinding effect was validated [14]. The deactivated EA apparatus was designed to show a same flashing light with the real EA apparatus, but no output of electric current. Though often used in clinical trials [39, 40], this deactivated EA control method has not yet been tested. An assessment of the effectiveness of blinding, by asking the patients which group they thought they were assigned to, was not done in this trial. It seems difficult that patients who have already received EA treatment in the past would not suspect that they are receiving sham treatment, even if they are told that they would receive low electrical stimulation treatment. Thus, the lack of validation on the blinding effect of the sham EA as a whole may overstate the treatment effect of EA group. The placebo acupuncture used in our trial, although without skin penetration, could still be perceived by participants. This perception was essential for successful participant blinding. Because the effect of acupuncture is closely related to the function of the nervous system, and a slight stimulus of gentle touch may play a role in pain inhibition by activating C tactile afferents [41], we could not exclude the possibility that sham EA had a curative effect. To implement a standardized operation among various patients, needles were lifted, thrusted and twirled evenly 3 times to achieve deqi, and no more manipulation was performed. We did not record how many patients in the EA and sham groups reported achieving deqi. For baseline data of the urine leakage measured by the 1-hour pad test, few outliers were only observed in the sham group. And imbalance of baseline SUI severity rated by the amount of urine leakage measured by the 1-hour pad test was observed: more severe SUI patients were in the sham EA group. The reason for these two questions were probably due to the small sample size, since patients were enrolled strictly according to inclusion criteria, and randomization was implemented via a central randomization system. Given that participants in this trial were mainly suffering from mild to moderate SUI at baseline, and we did not explore the effect of EA for various degrees of SUI severity, it was not clear which degree of SUI EA was most beneficial. Subgroup analysis based on SUI severity should be performed in a further large sample randomized placebo controlled study. The imputation method for missing data that we used, the last observation carried forward method, may result in bias and is less appropriate than multiple imputation [42, 43]. Though it is highly unlikely that it will make a difference in results if we use different methods for imputation, or even if we completely ignore missing data, since number of patients withdrawn are very low, and with similar number and reasons for drop-out across groups. In this trial, we only compared the effect of EA with that of the placebo control. We did not compare EA with the recommended first line treatment of PFMT. Therefore, the potential advantages such as fast onset of action and a long persistent therapeutic effect after stopping treatment still lack evidence, and will be studied in our next RCT study on the effect comparison of EA and PFMT. We only focused on AEs related to EA and did not record all the AEs, thus some AEs related to EA may have been omitted.

## Conclusions

EA may safely relieve urinary incontinence symptoms and improve quality of life in women with pure SUI. EA may have a specific treatment effect on pure SUI instead of a mere placebo effect. Since this is a pilot study, results should be interpreted with caution.

## Supporting Information

S1 CONSORT Checklist CONSORT Checklist. (DOC)

S1 Data Metadata. (XLSX)

S1 File Informed consent. (DOCX)

S2 File Case report form. (DOC)

S3 File Bladder diary. (DOC)

S1 Protocol Trial protocol. (DOCX)

## References

[1] Abrams P, Cardozo L, Fall M, Griffiths D, Rosier P, Ulmsten U, et al The standardisation of terminology in lower urinary tract function: report from the standardisation sub-committee of the International Continence Society. Urology. 2003;61:37–49. 12559262 10.1016/s0090-4295(02)02243-4

[2] Markland AD, Richter HE, Fwu CW, Eggers P, Kusek JW. Prevalence and trends of urinary incontinence in adults in the United States, 2001 to 2008. The Journal of urology. 2011;186:589–93. 10.1016/j.juro.2011.03.114 21684555 PMC3197263

[3] Zhu L, Lang J, Liu C, Han S, Huang J, Li X. The epidemiological study of women with urinary incontinence and risk factors for stress urinary incontinence in China. Menopause. 2009;16:831–6. 10.1097/gme.0b013e3181967b5d 19240656

[4] Lalos O, Berglund AL, Lalos A. Impact of urinary and climacteric symptoms on social and sexual life after surgical treatment of stress urinary incontinence in women: a long-term outcome. Journal of advanced nursing. 2001;33:316–27. 11251718 10.1046/j.1365-2648.2001.01667.x

[5] Swithinbank LV, Abrams P. The impact of urinary incontinence on the quality of life of women. World journal of urology. 1999;17:225–9. 10460405 10.1007/s003450050137

[6] Horng SS, Huang N, Wu SI, Fang YT, Chou YJ, Chou P. The epidemiology of urinary incontinence and it's influence on quality of life in Taiwanese middle-aged women. Neurourology and urodynamics. 2013;32:371–6. 10.1002/nau.22302 22972439

[7] Abrams P, Cardozo L, Khoury S, Wein A. INCONTINENCE (4th edition). Paris: Health Publication Ltd; 2009 Available: http://www.icud.info/PDFs/Incontinence.pdf.

[8] Lucas MG, Bosch RJ, Burkhard FC, Cruz F, Madden TB, Nambiar AK, et al European Association of Urology guidelines on assessment and nonsurgical management of urinary incontinence. Actas urologicas espanolas. 2013;37:199–213. 10.1016/j.acuro.2012.12.001 23452548

[9] Urinary incontinence: The management of urinary incontinence in women. 2013; Available: http://www.nice.org.uk/guidance/CG171/chapter/introduction.

[10] Bo K, Hilde G. Does it work in the long term?—A systematic review on pelvic floor muscle training for female stress urinary incontinence. Neurourology and urodynamics. 2013;32:215–23. 10.1002/nau.22292 22847318

[11] Zhao L, Wang SY. Efficacy impacts of the different treatment frequencies on female stress urinary incontinence. Zhongguo zhen jiu. 2013;33:1088–90. 24617234

[12] Bi W. Clinical study on electroacupuncture treatment of female stress incontinence. Chinese Archives of Traditional Chinese Medicine. 2007;25:1284–5.

[13] Wang Y, Zhishun L, Peng W, Zhao J, Liu B. Acupuncture for stress urinary incontinence in adults. Cochrane Database Syst Rev. 2013;7:CD009408 10.1002/14651858.CD009408.pub2 23818069 PMC11262557

[14] Liu B, Xu H, Ma R, Mo Q, Yan S, Liu Z. Effect of blinding with a new pragmatic placebo needle: a randomized controlled crossover study. Medicine. 2014;93:e200 10.1097/MD.0000000000000200 25501074 PMC4602803

[15] Pacific WROftW. WHO Standard Acupuncture Point Locations in the Western Pacific Region. Manila, Philippines 2008.

[16] Yin CS, Park HJ, Seo JC, Lim S, Koh HG. An Evaluation of the cun measurement system of acupuncture point location. The American journal of Chinese medicine. 2005;33:729–35. 16265985 10.1142/S0192415X05003284

[17] Hui KK, Nixon EE, Vangel MG, Liu J, Marina O, Napadow V, et al Characterization of the "deqi" response in acupuncture. BMC complementary and alternative medicine. 2007;7:33 17973984 10.1186/1472-6882-7-33 PMC2200650

[18] Abrams P, Blaivas JG, Stanton SL, Andersen JT. The standardisation of terminology of lower urinary tract function. The International Continence Society Committee on Standardisation of Terminology. Scandinavian journal of urology and nephrology Supplementum. 1988;114:5–19. 3201169

[19] Avery K, Donovan J, Peters TJ, Shaw C, Gotoh M, Abrams P. ICIQ: a brief and robust measure for evaluating the symptoms and impact of urinary incontinence. Neurourology and urodynamics. 2004;23:322–30. 15227649 10.1002/nau.20041

[20] Huang L, Zhang SW, Wu SL, Ma L, Deng XH. The Chinese version of ICIQ: a useful tool in clinical practice and research on urinary incontinence. Neurourology and urodynamics. 2008;27:522–4. 10.1002/nau.20546 18351586

[21] MacPherson H, Thomas K, Walters S, Fitter M. A prospective survey of adverse events and treatment reactions following 34,000 consultations with professional acupuncturists. Acupuncture in medicine. 2001;19:93–102. 11829165 10.1136/aim.19.2.93

[22] Mo Q, Ma X, Liu Z. Curative Effect Observation on the Treatment of Female Stress Urinary Incontinence with Electro-acupuncture. Beijing Journal of Traditional Chinese Medicine. 2013;32:434–6.

[23] Smither AR, Guralnick ML, Davis NB, See WA. Quantifying the natural history of post-radical prostatectomy incontinence using objective pad test data. BMC urology. 2007;7:2 17280607 10.1186/1471-2490-7-2 PMC1800860

[24] Sar D, Khorshid L. The effects of pelvic floor muscle training on stress and mixed urinary incontinence and quality of life. Journal of wound, ostomy, and continence nursing: official publication of The Wound, Ostomy and Continence Nurses Society / WOCN. 2009;36:429–35. 10.1097/WON.0b013e3181aaf539 19609165

[25] Liebergall-Wischnitzer M, Hochner-Celnikier D, Lavy Y, Manor O, Shveiky D, Paltiel O. Randomized trial of circular muscle versus pelvic floor training for stress urinary incontinence in women. J Womens Health (Larchmt). 2009;18:377–85. 19281321 10.1089/jwh.2008.0950

[26] Aksac B, Aki S, Karan A, Yalcin O, Isikoglu M, Eskiyurt N. Biofeedback and pelvic floor exercises for the rehabilitation of urinary stress incontinence. Gynecologic and obstetric investigation. 2003;56:23–7. 12867764 10.1159/000072327

[27] Sun MJ, Chang NE, Chen GD, Tsai HD. Comparison of suprapubic versus transobturator surgical treatments of female stress urinary incontinence. Taiwanese journal of obstetrics & gynecology. 2008;47:175–9. 18603502 10.1016/S1028-4559(08)60076-5

[28] Stamey TA. Endoscopic suspension of the vesical neck for urinary incontinence in females. Report on 203 consecutive patients. Annals of surgery. 1980;192:465–71. 7425693 10.1097/00000658-198010000-00005 PMC1346989

[29] Sjostrom M, Umefjord G, Stenlund H, Carlbring P, Andersson G, Samuelsson E. Internet-based treatment of stress urinary incontinence: a randomised controlled study with focus on pelvic floor muscle training. BJU international. 2013;112:362–72. 10.1111/j.1464-410X.2012.11713.x 23350826 PMC3798106

[30] Lin AT, Sun MJ, Tai HL, Chuang YC, Huang ST, Wang N, et al Duloxetine versus placebo for the treatment of women with stress predominant urinary incontinence in Taiwan: a double-blind, randomized, placebo-controlled trial. BMC urology. 2008;8:2 10.1186/1471-2490-8-2 18221532 PMC2266773

[31] Yalcin I, Peng G, Viktrup L, Bump RC. Reductions in stress urinary incontinence episodes: what is clinically important for women? Neurourology and urodynamics. 2010;29:344–7. 10.1002/nau.20744 19475576

[32] Hirakawa T, Suzuki S, Kato K, Gotoh M, Yoshikawa Y. Randomized controlled trial of pelvic floor muscle training with or without biofeedback for urinary incontinence. International urogynecology journal. 2013;24:1347–54. 10.1007/s00192-012-2012-8 23306768

[33] Gras S, Klarskov N, Lose G. Intraurethral injection of autologous minced skeletal muscle: a simple surgical treatment for stress urinary incontinence. The Journal of urology. 2014;192:850–5. 10.1016/j.juro.2014.04.005 24735937

[34] Chene G, Mansoor A, Jacquetin B, Mellier G, Douvier S, Sergent F, et al Female urinary incontinence and intravaginal electrical stimulation: an observational prospective study. European journal of obstetrics, gynecology, and reproductive biology. 2013;170:275–80. 10.1016/j.ejogrb.2013.06.011 23830965

[35] Yoshimura N, Miyazato M. Neurophysiology and therapeutic receptor targets for stress urinary incontinence. International journal of urology. 2012;19:524–37. 10.1111/j.1442-2042.2012.02976.x 22404481

[36] Noblett K, Siegel S, Mangel J, Griebling TL, Sutherland SE, Bird ET, et al Results of a prospective, multicenter study evaluating quality of life, safety, and efficacy of sacral neuromodulation at twelve months in subjects with symptoms of overactive bladder. Neurourology and urodynamics. 2014. 10.1002/nau.22707 25546568

[37] Wood LN, Anger JT. Urinary incontinence in women. BMJ. 2014;349:g4531 10.1136/bmj.g4531 25225003

[38] Ammi M, Chautard D, Brassart E, Culty T, Azzouzi AR, Bigot P. Transcutaneous posterior tibial nerve stimulation: evaluation of a therapeutic option in the management of anticholinergic refractory overactive bladder. International urogynecology journal. 2014;25:1065–9. 10.1007/s00192-014-2359-0 24599180

[39] Xue CC, Helme RD, Gibson S, Hogg M, Arnold C, Somogyi AA, et al Effect of electroacupuncture on opioid consumption in patients with chronic musculoskeletal pain: protocol of a randomised controlled trial. Trials. 2012;13:169 10.1186/1745-6215-13-169 22978476 PMC3495031

[40] Kim JH, Kim EJ, Seo BK, Lee S, Jung SY, Lee MH, et al Electroacupuncture for chemotherapy-induced peripheral neuropathy: study protocol for a pilot multicentre randomized, patient-assessor-blinded, controlled trial. Trials. 2013;14:254 10.1186/1745-6215-14-254 23945074 PMC3751258

[41] Olausson H, Wessberg J, Morrison I, McGlone F, Vallbo A. The neurophysiology of unmyelinated tactile afferents. Neuroscience and biobehavioral reviews. 2010;34:185–91. 10.1016/j.neubiorev.2008.09.011 18952123

[42] Sterne JA, White IR, Carlin JB, Spratt M, Royston P, Kenward MG, et al Multiple imputation for missing data in epidemiological and clinical research: potential and pitfalls. BMJ. 2009;338:b2393 10.1136/bmj.b2393 19564179 PMC2714692

[43] Mallinckrodt CH, Clark WS, David SR. Accounting for dropout bias using mixed-effects models. Journal of biopharmaceutical statistics. 2001;11:9–21. 11459446 10.1081/BIP-100104194

