# Supplementary material for: A Pilot Randomized Placebo Controlled Trial of Electroacupuncture for Women with Pure Stress Urinary Incontinence
Source: PLoS One. 2016 Mar 9;11(3):e0150821. doi: 10.1371/journal.pone.0150821 (PMC4784883; doi:10.1371/journal.pone.0150821)
Supplement: S1 File — (DOCX) [file pone.0150821.s003.docx]

**Informed consent**

Dear participants：

We sincerely invite you to participate in a clinical study on the evaluation of effectiveness and safety of acupuncture for women with pure stress urinary incontinence. This study is supported and funded by the “the 12th Five-Year Program’’ of the National Science and Technology Pillar Program (2012BAI24B01). The purpose of this study is to evaluate the effectiveness and safety of acupuncture for women with pure stress urinary incontinence and to assess the feasibility of the study protocol, thus to prepare for the further large sample multicenter randomized controlled clinical trial.

Before you decide to participate in the study, please read the following information carefully. It is helpful for you to know this study, understand why the study is performed, the study procedures, the duration and benefits of the study, risks and potential discomforts during and after study participation. If you prefer, you can also discuss this study with your relatives and friends, or consult medical doctors whom your trust for explanation and help to make the decision.

1. Study Introduction

Stress urinary incontinence (SUI) is a common disease for adult women. In china, the prevalence of urinary incontinence is approximately 46.5% among adult women, of which SUI accounts for approximately 59.6%. The prevalence of SUI increases with age. A prevalence up to 61.9% is observed for patients over 50 years. SUI negatively affects patients’ physical health and social and psychological well-being, however, it takes a long time for its routine treatments (life adjustment, behavior therapy, pelvic floor muscle training, etc) to take effect, and the patient compliance is not so good. Electroacupuncture(EA) can effectively manage urinary leakage of SUI in a short time, via improving the function of pelvic floor and external urethral muscle of SUI patients. To assess the efficacy of EA for women with SUI, twelve top hospitals in China (led by Guang’anmen hospital of Chinese academy of Chinese medical sciences), conduct this study jointly.

2. Things you need to do if participating in this study

(1) Doctors will ask and record your disease history, and carry out medical examination for you. You may need to take some tests (routine urine test, urodynamics, B ultrasound, 1-hour pad test, etc) and complete a 72-hour bladder diary to know whether you are eligible for this study.

(2) If you are eligible and willing to participate in the study, the study will go on according to the following steps.

a. You will be randomized into deep acupuncture or superficial acupuncture group according to the random number generated by the computer. The deep acupuncture and superficial acupuncture are two different methods which are both effective for SUI.

b. The duration of this study is 32 weeks, including a period of 2 weeks, a treatment period of 6 weeks, and a follow-up period of 24 weeks. The treatment will be given every other day, three sessions per week for 6 weeks, 18 sessions in total.

c. During the study period, you need to record bladder diaries and give it to your doctor on required time.

(3) Other requirements for your cooperation

As a participant of this study, you will have some relevant responsibilities, such as adherence to the schedule for examination, treatment, and outpatient follow-up. Additionally, you are also responsible for reporting any changes in your physical and mental status to your doctor during the study process regardless of whether you think these changes are related to the study or not.

You should follow the scheduled appointments with the doctor to come to the hospital for treatment (during follow-up, the doctor may get to know your conditions by phone or visiting your home). Your follow-up is very important because the doctor will determine whether the treatment that you are receiving really works, and the doctor will be able to guide the prevention and management of your symptoms timely.

3. Potential benefits of study participation

You may benefit from this study. The benefits may include improvement of symptoms. The study may also help doctors and researchers to further evaluate the efficacy of EA treatment for SUI. The information will be beneficial in the management of other patients with a similar condition in the future.

If you decide to participate in the study, you will get relevant physical, biochemical examination and the study intervention for free during the study period.

4. Potential side effects, risks, discomforts, and inconveniences

The doctors will make every effort to prevent and treat any side effects brought on by this study.

During acupuncture treatment, you may feel soreness, numbness, heavy, distension sensation, etc., which are normal reactions to acupuncture. Acupuncture treatment may have some adverse effects, but it is rare and mild. You may feel fainting due to your individual physique or emotional stress when receive acupuncture needling. Your symptoms should be relieved after the cessation of acupuncture treatment and rest. Bleeding, hematoma, and other phenomena may occur after acupuncture treatment, and these phenomena should disappear after applying local pressure. If infection occurs in the needle site, your doctor will handle it timely.

With the treatment following the study protocol in the study, if you experience adverse reactions and events related to acupuncture treatment, please feel free to call your doctor for help. The doctor will provide you timely treatment. If injuries have been confirmed and are caused by adverse reactions and events of the study, the study group will deal with them appropriately in accordance with relevant provisions.

If you experience any discomfort or new change of your symptoms, or any other unforeseen circumstances during study period, regardless of whether these events is relevant with treatment of the study or not, you shall promptly notify your doctor, and he / she will evaluate the condition and give you appropriate medical treatment.

5. Payments/compensation for participation

If you participate in the study, during the study, you will get relevant physical and biochemical examination and acupuncture treatment for free.

If adverse events occur during the study, medical experts committee will identify whether they are related to the study. The study group will deal with it appropriately in accordance with relevant provisions.

The treatment and examination required for your concomitant diseases will not be free of charge.

6. Confidentiality of personal information

All the information related to your participation in this study will be kept confidential by the institute where your participation takes place. Only the institutes responsible for the study, clinical research institutes, and ethics committees may have access to your medical records. Your name will not appear in any publications or reports related to this study.

We will make every effort to protect the privacy of your personal medical information as per legal requirements and laws.

7. How to acquire extra information?

You can ask any questions about the study at any time and will get answers timely.

If we notice any new information that may affect your willingness and decision to continue participating in the study, the doctor will keep you informed.

8. Can you voluntarily choose to participate in or withdraw from the study?

Whether to participate in this study or not entirely depends on your desire. You can refuse to participate in the study, or withdraw from the study at any time during the study, which will not affect the relationship between you and your doctor and will not affect your medical interests or interests in other areas.

For the consideration of your best interests, doctors or researchers may terminate your participation in this study at any time.

If you withdraw from the study for any reason, you may be asked for information related of acupuncture treatment or the use of other medications during your participation of the study. If the doctor considers it necessary, you may also be asked to have some laboratory tests and physical examinations performed.

9. What you need to do now?

Decide whether to participate in this study or not.

Before you make the decision to participate in the study, please ask your doctor if you have any concerns.

Thank you for reading the above information. If you decide to participate in this study, please tell your doctor, he / she will help you make arrangement for the study.

Please keep this document for your own record.

Statement of agreement

I have read the above information about this study and have the opportunity to discuss this study with my doctor and ask questions. All my questions were answered satisfactorily.

I understand the potential risks and benefits from participation in this study. I understand the participation of the study is voluntary and I confirm that I was given sufficient time for consideration of study participation. I confirm that I understand that:

I can always ask the doctor for additional/more information.

I can withdraw from the study at any time without discrimination or retaliation and my medical treatment and interests will not be affected.

I understand that if I withdraw from the study, I will tell the doctor the changes of my disease condition and complete the relevant physical and biochemical examinations if needed, which will be very helpful for the whole study.

If I need to take any other medications due to the changes of my medical condition, I will seek medical advice from the doctor beforehand or afterwards tell the doctor truthfully.

I agree to allow the research institute, collaborative institutes, and ethics committees to inspect the data relevant to my study participation.
I will receive a signed and dated copy of the informed consent form.

Finally, I decide and agree to participate in this study and ensure the adherence to doctor’s orders to the best I can.

Signature of patient Year month day

Telephone:

I confirm that I have explained this study in detail to the patient, including patient’s rights as well as the potential benefits and risks, and have given the patient a signed copy of the informed consent form.

Signature of doctor Year month day

Office phone number of doctor:
