# Supplementary material for: A Pilot Randomized Placebo Controlled Trial of Electroacupuncture for Women with Pure Stress Urinary Incontinence
Source: PLoS One. 2016 Mar 9;11(3):e0150821. doi: 10.1371/journal.pone.0150821 (PMC4784883; doi:10.1371/journal.pone.0150821)
Supplement: S2 File — (DOC) [file pone.0150821.s004.doc]

**A Pilot Randomized Placebo Controlled Trial of Electroacupuncture for Women with Pure Stress Urinary Incontinence**

**Case Report Form**

**VERSION 1.0_201201109**

**Random number：**|__|__|__|__|

**Initials of subject’s name：**|__|__|__|__|

**Doctor’s name：______________**

**Study period：2013/3 -2014/12**

**Flow chart of clinical research**

| **Period** | **Baseline** | **Treatment** | | | **Follow-up** | | | | | | | |
| --- | --- | --- | --- | --- | --- | --- | --- | --- | --- | --- | --- | --- |
| **Visit** | **1** | **2** | **3** | **4** | **5** | **6** | **7** | **8** | **9** | **10** | **11** | **12** |
| **Time (week)** | Week0 | Week2 | Week4 | Week6 | Week15 | Week16 | Week17 | Week18 | Week27 | Week28 | Week29 | Week30 |
| Sign informed consent | **X** |  |  |  |  |  |  |  |  |  |  |  |
| Inclusion/exclusion criteria | **X** |  |  |  |  |  |  |  |  |  |  |  |
| Demography data | **X** |  |  |  |  |  |  |  |  |  |  |  |
| History of SUI | **X** |  |  |  |  |  |  |  |  |  |  |  |
| Routine urine test | **X** |  |  |  |  |  |  |  |  |  |  |  |
| Urine flow rate | **X** |  |  |  |  |  |  |  |  |  |  |  |
| Residual urine volume by bladder ultrasound | **X** |  |  |  |  |  |  |  |  |  |  |  |
| Amount of urine leakage measured by the 1-hour pad test | **X** | **X** |  | **X** |  |  |  |  |  |  |  |  |
| 72 IEF | **X** | **X** | **X** | **X** | **X** | **X** | **X** | **X** | **X** | **X** | **X** | **X** |
| ICIQ-SF questionnaire | **X** |  | **X** | **X** |  |  |  | **X** |  |  |  | **X** |
| Patient self-evaluation of therapeutic effect |  | **X** | **X** | **X** |  |  |  | **X** |  |  |  | **X** |
| Adverse events | **X** | | | | | | | | | | | |

**Visit 1: Baseline (Week 0)**

**Visit Date:** Day|__|__| Month|__|__| Year 201|__|

**Date for signing informed consent：**Day|__|__| Month|__|__| Year 201|__|

| **Inclusion criteria** | **Yes** | **No** |
| --- | --- | --- |
| 1. women diagnosed as SU; | □**_1_** | □**_0_** |
| 1. aged 40 to 75 years; | □**_1_** | □**_0_** |

| **Exclusion criteria** | **否** | **是** |
| --- | --- | --- |
| 1. other type of UI (urge, mixed, or overflow UI, etc); | □**_0_** | □**_1_** |
| 1. symptomatic urinary tract infection; | □**_0_** | □**_1_** |
| 1. ever received UI or pelvic surgery; | □**_0_** | □**_1_** |
| 1. severe pelvic organ prolapse ≥ degree 2; | □**_0_** | □**_1_** |
| 1. residual urinary volume >30 ml; | □**_0_** | □**_1_** |
| 1. maximum flow rate ≤ 20 ml/s; | □**_0_** | □**_1_** |
| 1. limited in walking, stairs climbing and running; | □**_0_** | □**_1_** |
| 1. receiving specialized treatment for SUI or use of medicine affecting bladder function; | □**_0_** | □**_1_** |
| 1. serious cardiovascular, cerebral, liver, kidney, or psychiatric disease, diabetes, multiple system atrophy, injury of cauda equina, or myeleterosis; | □_0_ | □_1_ |
| 1. being pregnant or breastfeeding; | □_0_ | □_1_ |
| 1. with cardiac pacemaker, metal allergy or severe needle phobia; | □_0_ | □_1_ |
| 1. unlikely to give written informed consent. | □_0_ | □_1_ |

| **Demography data** | | | | | |
| --- | --- | --- | --- | --- | --- |
| **Gender** | Female | **Birthday** | Day|__|__| Month|__|__| Year |__|__|__|__| | | |
| **Educational level** | □**_1_** Primaryeducation or below □**_2_** Secondary education □**_3_** Tertiary education | | | | |
| **Childbearing** | □**_0_** No | | | | |
|  | □**_1_** Yes, Initial childbearing age \|__\|__\|; Number of births \|__\|; Number of vaginal deliveries; Number of cesarean \|__\| | | | | |
| **Menopause** | □**_1_** Yes □**_0_** No | | | Hysterectomy | □**_1_** Yes □**_0_** No |
| **BMI** | |__|__|.|__| | | | Phone Number |  |

| **History of SUI** | |
| --- | --- |
| **Duration** | |__|__|Year |__|__| Month |
| **SUI degree** | □**_1_**Mild □**_2_** Moderate □**_3_** Severe |
| **SUI Treatment** | □**_0_** No □**_1_**Yes |

| **Examination** |
| --- |
| **Routine urine test Date:** Day|__|__| Month|__|__| Year |__|__|__|__| |
| □**_0_** Not done □**_1_** Normal □**_2_** Abnormal, without clinical significance □**_3_** Abnormal, with clinical significance |
| **Urine flow rate Date:** Day|__|__| Month|__|__| Year |__|__|__|__| |
| □**_0_** Not done □_1_ |__|__| ml/s |
| **Residual urine volume Date:** Day|__|__| Month|__|__| Year |__|__|__|__| |
| □**_0_** Not done □_1_|__|__| ml |

| **Therapeutic evaluation** | |
| --- | --- |
| **1. Amount of urine leakage measured by the 1-hour pad test:** |__|__|.|__| **g** | |
| **2. Based on 72-hour bladder diary** | **72-hour IEF**：|__|__| |
|  | **SUI degree：**□**_1_** Mild □**_2_** Moderate □**_3_** Severe |

| **ICIQ-SF questionnaire** |
| --- |
| Many people leak urine some of the time. We are trying to find out how many people leak urine, and how much this bothers them. We would be grateful if you could answer the following questions, thinking about how you have been, on average, over the PAST FOUR WEEKS. |
| 1．How often do you leak urine? (Tick one box)  never □ 0  about once a week or less often □ 1  two or three times a week □ 2  about once a day □ 3  several times a day □ 4  all the time □ 5 |
| We would like to know how much you think leaks.  2． How much urine do you usually leak (whether you wear protection or not)?  None □ 0  a small amount □ 2  a moderate amount □ 4  a large amount □ 6 |
| 3．Overall, how much does leaking urine interfere with your everyday life?  Please ring a number between 0 (not at all) and 10 (a great deal)  0 1 2 3 4 5 6 7 8 9 10  not at all a great deal |
| 4. When does urine leak? (please tick all that apply to you)  never-urine does not leak □  leaks before you can get to the toilet □  leaks when you cough or sneeze □  leaks when you are asleep □  leaks when you are physically active/exercising □  leaks when you have finished urinaring and are dressed □  leaks all the time □ |

**Visit 2: Treatment (Week 2)**

**Visit Date:** Day|__|__| Month|__|__| Year 201|__|

| **Therapeutic evaluation** | |
| --- | --- |
| **1. Amount of urine leakage measured by the 1-hour pad test:** |__|__|.|__| **g** | |
| **2. Based on 72-hour bladder diary** | **72-hour IEF**：|__|__| |
|  | **SUI degree：**□**_1_** Mild □**_2_** Moderate □**_3_** Severe |

**Visit 3: Treatment (Week 4)**

**Visit Date:** Day|__|__| Month|__|__| Year 201|__|

| **Therapeutic evaluation** | |
| --- | --- |
| **1. Amount of urine leakage measured by the 1-hour pad test:** |__|__|.|__| **g** | |
| **2. Based on 72-hour bladder diary** | **72-hour IEF**：|__|__| |
|  | **SUI degree：**□**_1_** Mild □**_2_** Moderate □**_3_** Severe |

| **ICIQ-SF questionnaire** |
| --- |
| Many people leak urine some of the time. We are trying to find out how many people leak urine, and how much this bothers them. We would be grateful if you could answer the following questions, thinking about how you have been, on average, over the PAST FOUR WEEKS. |
| 1．How often do you leak urine? (Tick one box)  never □ 0  about once a week or less often □ 1  two or three times a week □ 2  about once a day □ 3  several times a day □ 4  all the time □ 5 |
| We would like to know how much you think leaks.  2． How much urine do you usually leak (whether you wear protection or not)?  None □ 0  a small amount □ 2  a moderate amount □ 4  a large amount □ 6 |
| 3．Overall, how much does leaking urine interfere with your everyday life?  Please ring a number between 0 (not at all) and 10 (a great deal)  0 1 2 3 4 5 6 7 8 9 10  not at all a great deal |
| 4. When does urine leak? (please tick all that apply to you)  never-urine does not leak □  leaks before you can get to the toilet □  leaks when you cough or sneeze □  leaks when you are asleep □  leaks when you are physically active/exercising □  leaks when you have finished urinaring and are dressed □  leaks all the time □ |

**Visit 4: Treatment (Week 6)**

**Visit Date:** Day|__|__| Month|__|__| Year 201|__|

| **Therapeutic evaluation** | |
| --- | --- |
| **1. Amount of urine leakage measured by the 1-hour pad test:** |__|__|.|__| **g** | |
| **2. Based on 72-hour bladder diary** | **72-hour IEF**：|__|__| |
|  | **SUI degree：**□**_1_** Mild □**_2_** Moderate □**_3_** Severe |

**Visit 5: Follow up (Week 15)**

**Visit Date:** Day|__|__| Month|__|__| Year 201|__|

| **Therapeutic evaluation** | |
| --- | --- |
| **1. Amount of urine leakage measured by the 1-hour pad test:** |__|__|.|__| **g** | |
| **2. Based on 72-hour bladder diary** | **72-hour IEF**：|__|__| |
|  | **SUI degree：**□**_1_** Mild □**_2_** Moderate □**_3_** Severe |

**Visit 6: Follow up (Week 16)**

**Visit Date:** Day|__|__| Month|__|__| Year 201|__|

| **Therapeutic evaluation** | |
| --- | --- |
| **1. Amount of urine leakage measured by the 1-hour pad test:** |__|__|.|__| **g** | |
| **2. Based on 72-hour bladder diary** | **72-hour IEF**：|__|__| |
|  | **SUI degree：**□**_1_** Mild □**_2_** Moderate □**_3_** Severe |

**Visit 7: Follow up (Week 17)**

**Visit Date:** Day|__|__| Month|__|__| Year 201|__|

| **Therapeutic evaluation** | |
| --- | --- |
| **1. Amount of urine leakage measured by the 1-hour pad test:** |__|__|.|__| **g** | |
| **2. Based on 72-hour bladder diary** | **72-hour IEF**：|__|__| |
|  | **SUI degree：**□**_1_** Mild □**_2_** Moderate □**_3_** Severe |

**Visit 8: Follow up (Week 18)**

**Visit Date:** Day|__|__| Month|__|__| Year 201|__|

| **Therapeutic evaluation** | |
| --- | --- |
| **1. Amount of urine leakage measured by the 1-hour pad test:** |__|__|.|__| **g** | |
| **2. Based on 72-hour bladder diary** | **72-hour IEF**：|__|__| |
|  | **SUI degree：**□**_1_** Mild □**_2_** Moderate □**_3_** Severe |

| **ICIQ-SF questionnaire** |
| --- |
| Many people leak urine some of the time. We are trying to find out how many people leak urine, and how much this bothers them. We would be grateful if you could answer the following questions, thinking about how you have been, on average, over the PAST FOUR WEEKS. |
| 1．How often do you leak urine? (Tick one box)  never □ 0  about once a week or less often □ 1  two or three times a week □ 2  about once a day □ 3  several times a day □ 4  all the time □ 5 |
| We would like to know how much you think leaks.  2． How much urine do you usually leak (whether you wear protection or not)?  None □ 0  a small amount □ 2  a moderate amount □ 4  a large amount □ 6 |
| 3．Overall, how much does leaking urine interfere with your everyday life?  Please ring a number between 0 (not at all) and 10 (a great deal)  0 1 2 3 4 5 6 7 8 9 10  not at all a great deal |
| 4. When does urine leak? (please tick all that apply to you)  never-urine does not leak □  leaks before you can get to the toilet □  leaks when you cough or sneeze □  leaks when you are asleep □  leaks when you are physically active/exercising □  leaks when you have finished urinaring and are dressed □  leaks all the time □ |

**Visit 9: Follow up (Week 27)**

**Visit Date:** Day|__|__| Month|__|__| Year 201|__|

| **Therapeutic evaluation** | |
| --- | --- |
| **1. Amount of urine leakage measured by the 1-hour pad test:** |__|__|.|__| **g** | |
| **2. Based on 72-hour bladder diary** | **72-hour IEF**：|__|__| |
|  | **SUI degree：**□**_1_** Mild □**_2_** Moderate □**_3_** Severe |

**Visit 10: Follow up (Week 28)**

**Visit Date:** Day|__|__| Month|__|__| Year 201|__|

| **Therapeutic evaluation** | |
| --- | --- |
| **1. Amount of urine leakage measured by the 1-hour pad test:** |__|__|.|__| **g** | |
| **2. Based on 72-hour bladder diary** | **72-hour IEF**：|__|__| |
|  | **SUI degree：**□**_1_** Mild □**_2_** Moderate □**_3_** Severe |

**Visit 11: Follow up (Week 29)**

**Visit Date:** Day|__|__| Month|__|__| Year 201|__|

| **Therapeutic evaluation** | |
| --- | --- |
| **1. Amount of urine leakage measured by the 1-hour pad test:** |__|__|.|__| **g** | |
| **2. Based on 72-hour bladder diary** | **72-hour IEF**：|__|__| |
|  | **SUI degree：**□**_1_** Mild □**_2_** Moderate □**_3_** Severe |

**Visit 12: Follow up (Week 30)**

**Visit Date:** Day|__|__| Month|__|__| Year 201|__|

| **Therapeutic evaluation** | |
| --- | --- |
| **1. Amount of urine leakage measured by the 1-hour pad test:** |__|__|.|__| **g** | |
| **2. Based on 72-hour bladder diary** | **72-hour IEF**：|__|__| |
|  | **SUI degree：**□**_1_** Mild □**_2_** Moderate □**_3_** Severe |

| **ICIQ-SF questionnaire** |
| --- |
| Many people leak urine some of the time. We are trying to find out how many people leak urine, and how much this bothers them. We would be grateful if you could answer the following questions, thinking about how you have been, on average, over the PAST FOUR WEEKS. |
| 1．How often do you leak urine? (Tick one box)  never □ 0  about once a week or less often □ 1  two or three times a week □ 2  about once a day □ 3  several times a day □ 4  all the time □ 5 |
| We would like to know how much you think leaks.  2． How much urine do you usually leak (whether you wear protection or not)?  None □ 0  a small amount □ 2  a moderate amount □ 4  a large amount □ 6 |
| 3．Overall, how much does leaking urine interfere with your everyday life?  Please ring a number between 0 (not at all) and 10 (a great deal)  0 1 2 3 4 5 6 7 8 9 10  not at all a great deal |
| 4. When does urine leak? (please tick all that apply to you)  never-urine does not leak □  leaks before you can get to the toilet □  leaks when you cough or sneeze □  leaks when you are asleep □  leaks when you are physically active/exercising □  leaks when you have finished urinaring and are dressed □  leaks all the time □ |

| **Drug combination:** □**_0_** No; □**_1_** Yes, please continue to fill in the following form | | | | | | | | | | | |
| --- | --- | --- | --- | --- | --- | --- | --- | --- | --- | --- | --- |
| **No.** | **Drug name** | **Indication** | **Due to AEs?** | **Dosage form** | **Dosage** | **Dose unit** | **Administration frequency** | **Administration route** | **Start date** | **still last when the trial finished?** | |
|  |  |  |  |  |  |  |  |  |  | **Yes** | **No→Endign date** |
| **1** |  |  | □**_0_** No □**_1_** Yes,  AE No.|__|__| |  |  |  |  |  |  | □**_1_** |  |
| **2** |  |  | □**_0_** No □**_1_** Yes,  AE No.|__|__| |  |  |  |  |  |  | □**_1_** |  |
| **3** |  |  | □**_0_** No □**_1_** Yes,  AE No.|__|__| |  |  |  |  |  |  | □**_1_** |  |
| **4** |  |  | □**_0_** No □**_1_** Yes,  AE No.|__|__| |  |  |  |  |  |  | □**_1_** |  |
| **6** |  |  | □**_0_** No □**_1_** Yes,  AE No.|__|__| |  |  |  |  |  |  | □**_1_** | | |
| **7** |  |  | □**_0_** No □**_1_** Yes,  AE No.|__|__| |  |  |  |  |  |  | □**_1_** |  |
| **8** |  |  | □**_0_** No □**_1_** Yes,  AE No.|__|__| |  |  |  |  |  |  | □**_1_** |  |
| **9** |  |  | □**_0_** No □**_1_** Yes,  AE No.|__|__| |  |  |  |  |  |  | □**_1_** |  |
| **10** |  |  | □**_0_** No □**_1_** Yes,  AE No.|__|__| |  |  |  |  |  |  | □**_1_** |  |

| **Adverse events:** □**_0_** No; □**_1_** Yes, please continue to fill in the following form | | | | | | | | | | | |
| --- | --- | --- | --- | --- | --- | --- | --- | --- | --- | --- | --- |
| **No.** | **AE** | **Start date** | **still last when the trial finished?** | | **Severity** | **related with treatment?** | **Measures taken for treatment?** | **Other measures** | **Outcome** | **Is it SAE?** | |
|  |  |  | **Yes** | **No→Endign date** |  |  |  |  |  | **No** | **Yes, type of SAE and report date** |
| **1** |  |  | □**_1_** |  | □**_1_**Mild  □**_2_** Moderate  □**_3_** Severe |  |  |  |  | □**_0_** |  |
| **2** |  |  | □**_1_** |  | □**_1_**Mild  □**_2_** Moderate  □**_3_** Severe |  |  |  |  | □**_0_** |  |
| **3** |  |  | □**_1_** |  | □**_1_**Mild  □**_2_** Moderate  □**_3_** Severe |  |  |  |  | □**_0_** |  |
| **4** |  |  | □**_1_** |  | □**_1_**Mild  □**_2_** Moderate  □**_3_** Severe |  |  |  |  | □**_0_** |  |
| **5** |  |  | □**_1_** |  | □**_1_**Mild  □**_2_** Moderate  □**_3_** Severe |  |  |  |  | □**_0_** |  |
| **6** |  |  | □**_1_** |  | □**_1_**Mild  □**_2_** Moderate  □**_3_** Severe |  |  |  |  | □**_0_** |  |
| **7** |  |  | □**_1_** |  | □**_1_**Mild  □**_2_** Moderate  □**_3_** Severe |  |  |  |  | □**_0_** |  |

| **Summary** |
| --- |
| Date for the first treatment: Day|__|__| Month|__|__| Year 201|__|  Date for the last treatment: Day|__|__| Month|__|__| Year 201|__|  Does the patient complete the whole trial?  □**_1_** Yes;  □**_0_** No; Date for trial suspending: Day|__|__| Month|__|__| Year 201|__| |
| **Reasons for trial suspending (choose the most important one)** |
| □**_1_** Suspended by DSMB  □**_2_** Violate trial protocol  □**_3_** Adverse events  □**_4_** For safety of patient, researcher stops the treatment though no adverse event occures  □**_5_** Lack treatment effect  □**_6_** Withdraw by patient  □**_7_** Lost to follow up  □**_8_** Be pregnant  □**_9_** die  □**_10_** Other reasons |
