# Supplementary material for: A Pilot Randomized Placebo Controlled Trial of Electroacupuncture for Women with Pure Stress Urinary Incontinence
Source: PLoS One. 2016 Mar 9;11(3):e0150821. doi: 10.1371/journal.pone.0150821 (PMC4784883; doi:10.1371/journal.pone.0150821)
Supplement: S3 File — (DOC) [file pone.0150821.s005.doc]

**Bladder Diary**

**Date (Year/month/day):**

| **Urinary incontinence** | **Liquid intake** |
| --- | --- |
| time/frequency/state | time/type/amount(ml) |
| **Morning 6:00** | **Morning 6:00** |
| **Example** 6:23/once/get out of bed | **Example** 6:50/1 cup of water/100ml |
|  |  |
|  |  |
|  |  |
|  |  |
| **Noon 12:00** | **Noon 12:00** |
|  |  |
|  |  |
|  |  |
|  |  |
| **Afternoon 18:00** | **Afternoon 18:00** |
|  |  |
|  |  |
|  |  |
|  |  |
| **Midnight 12:00** | **Midnight 12:00** |
|  |  |
|  |  |
|  |  |
|  |  |
| **Please record the number of urinal pad you use today:** | |
