# Supplementary material for: A Pilot Randomized Placebo Controlled Trial of Electroacupuncture for Women with Pure Stress Urinary Incontinence
Source: PLoS One. 2016 Mar 9;11(3):e0150821. doi: 10.1371/journal.pone.0150821 (PMC4784883; doi:10.1371/journal.pone.0150821)
Supplement: S1 Protocol — (DOCX) [file pone.0150821.s006.docx]

**Efficacy study of electroacupuncture for women with pure stress urinary incontinence: study protocol for a pilot randomized placebo controlled trial**

**Background**

Acupuncture is a potential conservative therapy for women with stress urinary incontinence (SUI). There are limited evidences to support its effectiveness due to poor quality of existing studies.

**Objective**

To explore the efficacy and safety of electroacupuncture (EA) for women with pure SUI.

**Methods**

**Study design**

This is a randomized, placebo-controlled study comparing EA with sham EA. Eighty women with pure SUI will be recruited from department of acupuncture and moxibustion of Guang’anmen hospital of China Academy of Chinese Medical Sciences (CACMS), Beijing, China. Participants will be randomly assigned to EA group or sham EA group by central randomization with a 1:1 ratio. The central randomization system was performed by the Clinical Evaluation Center of CACMS in Beijing. Random number and group assignment will be offered by telephone, mobile phone, or website from the Clinical Evaluation Center. In this study, participants, outcome assessors and statisticians are blinded to treatment allocation. This study was registered on ClinicalTrials.gov (Identifier: NCT02445573).

**Ethics**

The study was conducted in accordance with the Declaration of Helsinki, and the protocol was reviewed and approved by the Ethics Committee of Guang’anmen hospital of China Academy of Chinese Medical Sciences (CACMS). Written informed consent will be obtained from each subject before patients entering the trial.

**Participants**

The trial is planned to recruit 80 women with pure SUI. Potential participants were guided to finish the laboratory examinations (urine routine, urine flow rate and the residual urine), fill out 72-hour bladder diary and ICIQ-SF, and take 1 hour pad test by research assistants during 1-week baseline assessment to determine eligibility.

Diagnostic criteria are as follows:

1. involuntary urine leakage on effort, exertion, sneezing or coughing, which stopped when the stress ends;
2. visible involuntary leakage from the urethra synchronous with increased abdominal pressure;
3. a pad weight gain >1 g in 1-hour pad test.

Inclusion criteria are as follows:

1. 40 to 75 years of age;
2. meet diagnostic criteria;
3. without symptoms of urinary frequency and urgency.

Exclusion criteria are as follows:

1. met any of the following criteria: other type of UI (urge, mixed, or overflow UI, etc);
2. symptomatic urinary tract infection; ever received UI or pelvic surgery; a severity of pelvic organ prolapse ≥ degree 2;
3. residual urinary volume >30 ml;
4. maximum flow rate ≤ 20 ml/s;
5. limited in walking, stairs climbing and running; receiving specialized treatment for SUI, or taking medicine affecting bladder function;
6. serious cardiovascular, cerebral, liver, kidney, or psychiatric disease, diabetes, multiple system atrophy, injury of cauda equina, or myeleterosis;
7. being pregnant or breastfeeding;
8. with cardiac pacemaker, metal allergy or severe needle phobia;
9. unlike to give written formed consent.

**Interventions**

Acupuncture operation will be performed by registered acupuncturists who have more than two years’ experience.

Disposable acupuncture needle (size 0.30×75 mm), pragmatic placebo needle (size 0.30×25 mm) and SDZ-V EA apparatus (all were Hwato Brand, Suzhou Medical Appliance Factory, Suzhou, China) were used in this trial.

***EA group***

In EA group, acupoints of bilateral BL33 (Zhongliao) and BL35 (Huiyang) were used. BL33 is on the third posterior sacral foremen. BL35 is 0.5 cun lateral to the tip of the coccyx. When acupuncturing, adhesive pads were first pasted on acupoints after sterilization in either group. In EA group, participants were needled at bilateral BL33 at an angle of 30 to 45 degree inward and downward, and at bilateral BL35 slightly toward upside and outside, to a depth of 50 to 60 mm using acupuncture needles of size 0.30×75 mm. Needles were then lifted, thrusted and twirled evenly for 3 times to achieve deqi (also termed as needle sensation, is a composite of unique sensations interpreted as the flow of qi induced by acupuncture, and is believed to be essential for a good clinical effect). To implement a standardized operation among different participants, no more needle manipulation would be performed even if the required manipulation failed to achieve deqi. Paired electrodes of EA apparatus were attached transversely to bilateral BL33 and BL35 (using real electrodes) respectively, with a continuous wave of 50 Hz and a current intensity of 1-5 mA for 30 min. Participants were treated with EA 3 sessions a week on alternate days for 6 successive weeks.

***sham EA group***

In sham EA group, participants were needled at sham BL33 and sham BL35, which were about 20 mm lateral to BL33 and BL35, respectively, with blunt needle tips piercing adhesive pads and not piercing the surface of the skin, using placebo needles of size 0.30×25 mm. Needles were then lifted, thrusted and twirled evenly for 3 times to achieve deqi. Paired electrodes of EA apparatus were attached transversely to bilateral sham BL33 and sham BL35 (using sham electrodes) respectively with same parameters and treatment courses.

To improve the effect of blindness, participants were informed that this trial was to compare the effects of two EA methods for pure SUI. One was traditional EA using conventional acupoints and electric current, and the other was non-traditional EA by needling on non-traditional acupoints with a weak electric current. The inform was approved by our ethics committee. Participants were discouraged from any other specialized treatments of SUI, mainly referring to SUI medications such as duloxetine, PFMT, feedback therapy, electrical or magnetic stimulation via pelvic floor, vagina or anus, and transcutaneous electrical nerve stimulation to pelvic floor. During the treatment period, if a participant was in her menstrual cycle, the treatment was postponed until the cycle ended. The length of delay was not included in the treatment period. It took 32 weeks in total for a patient to complete the trial: 1 week’s screening, 1 week’s baseline assessment, 6 weeks’ treatment, and 24 weeks’ follow up.

**Outcome measures**

**Primary outcome:**

Change from baseline of urine leakage after 6 weeks measured by 1-hour pad test.

**Second outcomes:**

1. Change from baseline of the mean 72-hour incontinence episode frequency (IEF) during weeks 1-6, weeks 15-18 and weeks 27-30;
2. Change from baseline of the total ICIQ-SF scores at weeks 6, 18 and 30;
3. Patient self-evaluation of therapeutic effect at weeks 6, 18 and 30.

Data of IEF was from 72-hour bladder diary. The bladder diary was recorded in detail the time and frequency of UI, activity that occurred at the time of leak, and the type and volume of liquid intake. In case that a participant was in her menstrual cycle or suffering from severe cough during an assessment week, she needed not to record the bladder diary until the end of cycle or the recovery of cough.

**Safety Monitoring**

We only recorded and evaluated all the serious adverse events (SAEs) and the AEs related with EA, including local pain from needling, slight bleeding or hematoma, persistent pain after EA, discomfort after EA (remaining uncomfortable needle sensation, fatigue, etc), stuck needle, fainting during acupuncture, broken needle, and infection. Participants were instructed to report AEs throughout the study by contacting the outcome assessor at each visit or by telephone.

Pain or discomfort caused by acupuncture (if there was) was assessed using a 10-point visual analogue scale (VAS, 0 indicates no pain/discomfort, and 10 indicates the severest pain/discomfort). Acupuncture pain with a spontaneously remission within 30 min after acupuncture was not regarded as AE. Other common AEs which were obviously not related to acupuncture (like catching cold) were neglected. The rate of AEs were calculated using data from patients with ≥1 AEs.

**Sample Size and Statistical Analysis**

Assuming a two-sided alpha of 0.05, power of 90%, and a 20% drop-out, a sample size of 36 would be needed for each group to detect a between-group mean difference of 1.46 g (SD 1.74) in the reduction from baseline of urine leakage measured by 1-hour pad test according to results of our previous SUI study comparing deep EA with shallow EA[[19](#_ENREF_19)]. We expanded the sample size to 80 cases (40 cases per group) to increase the reliability of the study.

We performed statistical analysis based on the intention-to-treat principle. All patients accepting randomization were included in the analysis. Missing data were filled in by the last observed value. Continuous data were presented with mean and standard deviation [M (SD)] if they were normally distributed, or with median and interquartile range [Median (IQR)] if they were were abnormally distributed. Categorical variables were expressed as numbers and percentages. Student t tests or Mann-Whitney U tests were used for the comparison of continuous variables; chi-square tests, Fisher’s exact tests or Kruskal-Wallis H tests were used to compare categorical variables, as appropriate. For measures collected at two time points, paired t-tests or Wilcoxon signed rank tests were used as appropriate. A statistically significant difference was set at P<0.05. All statistical analyses were performed using SPSS statistical software (version 20.0, International Business Machines Corporation, China).
